# Does filter-aided sample preparation provide sufficient method linearity for quantitative plant shotgun proteomics?

**DOI:** 10.3389/fpls.2022.874761

**Published:** 2022-11-23

**Authors:** Tatiana Leonova, Christian Ihling, Mohamad Saoud, Nadezhda Frolova, Robert Rennert, Ludger A. Wessjohann, Andrej Frolov

**Affiliations:** ^1^ Department of Bioorganic Chemistry, Leibniz Institute of Plant Biochemistry, Halle (Saale), Germany; ^2^ Department of Biochemistry, St Petersburg State University, St Petersburg, Russia; ^3^ Institute of Pharmacy, Department of Pharmaceutical Chemistry and Bioanalytics, Martin-Luther Universität Halle-Wittenberg, Halle (Saale), Germany

**Keywords:** detergent-assisted proteolysis, filter aided sample preparation (FASP), label-free quantification, LC-MS, phenol extraction, plant proteomics, shotgun proteomics, sodium dodecyl sulfate

## Abstract

Due to its outstanding throughput and analytical resolution, gel-free LC-based shotgun proteomics represents the gold standard of proteome analysis. Thereby, the efficiency of sample preparation dramatically affects the correctness and reliability of protein quantification. Thus, the steps of protein isolation, solubilization, and proteolysis represent the principal bottleneck of shotgun proteomics. The desired performance of the sample preparation protocols can be achieved by the application of detergents. However, these compounds ultimately compromise reverse-phase chromatographic separation and disrupt electrospray ionization. Filter-aided sample preparation (FASP) represents an elegant approach to overcome these limitations. Although this method is comprehensively validated for cell proteomics, its applicability to plants and compatibility with plant-specific protein isolation protocols remain to be confirmed. Thereby, the most important gap is the absence of the data on the linearity of underlying protein quantification methods for plant matrices. To fill this gap, we address here the potential of FASP in combination with two protein isolation protocols for quantitative analysis of pea (*Pisum sativum*) seed and *Arabidopsis thaliana* leaf proteomes by the shotgun approach. For this aim, in comprehensive spiking experiments with bovine serum albumin (BSA), we evaluated the linear dynamic range (LDR) of protein quantification in the presence of plant matrices. Furthermore, we addressed the interference of two different plant matrices in quantitative experiments, accomplished with two alternative sample preparation workflows in comparison to conventional FASP-based digestion of cell lysates, considered here as a reference. The spiking experiments revealed high sensitivities (LODs of up to 4 fmol) for spiked BSA and LDRs of at least 0.6 × 10^2^. Thereby, phenol extraction yielded slightly better recoveries, whereas the detergent-based method showed better linearity. Thus, our results indicate the very good applicability of FASP to quantitative plant proteomics with only limited impact of the protein isolation technique on the method’s overall performance.

## Introduction

To date, bottom-up proteomics represents one of the most established methodological platforms for post-genomic research (Zhang et al., 2013). During the last decade, gel-free LC-based shotgun proteomics became a gold standard of proteome analysis due to its higher throughput, superior proteome coverage, better analytical resolution, and reproducibility (Smolikova et al., 2020). However, because of the mechanistic limitations of electrospray ionization (ESI), shotgun proteomics is critically sensitive to detergents (Frolov et al., 2018). On the other hand, in contrast to in-gel proteolysis (which can be quantitatively accomplished in ammonium bicarbonate buffer), in-solution digestion for shotgun proteomics ultimately requires supplementation of detergents to ensure quantitative solubilization of protein isolates and their efficient proteolysis (Wiśniewski et al., 2009). Therefore, a broad range of protocols for detergent-assisted proteolysis, employing degradable or selectively removable detergents, were successfully established to date (Waas et al., 2014). One of the most widely spread detergent-based digestion techniques is the filter-aided sample preparation (FASP)—an elegant approach efficiently combining the advantages of in-gel and in-solution digestion protocols (Wiśniewski et al., 2009). This method allows complete solubilization of dried protein isolates in sodium dodecyl sulfate (SDS) aqueous solutions, centrifugal concentration of reconstituted proteins, efficient reduction, and alkylation, followed by detergent removal and digestion of proteins in one centrifugal filter device. After the introduction of FASP by Mann’s group in 2009 (Wiśniewski et al., 2009), the original protocol was subjected to various modifications to improve recovery of proteolytic peptides (Wiśniewski, 2019). Thus, binding of peptides to the membrane could be reduced by conditioning of filters with 5% (*v/v*) Tween^®^ 20, whereas supplementation of 0.2% (*w/v*) deoxycholic acid prior to proteolysis resulted in enhancement of digestion efficiency (Erde et al., 2014). Furthermore, implementation of a multienzyme digestion FASP provided improved protein identification rates and sequence coverage of individual species (Wiśniewski and Mann, 2012).

The principal advantage of FASP technology is its ability to couple uniform and efficient protein extraction and/or reconstitution protocols with powerful proteolysis techniques. In this context, due to the tremendous variation in the properties of biological matrices from different (plant) species, the entire sample preparation protocol typically requires intensive optimization for each of them to ensure the best possible performance (Wang et al., 2018). For this reason, integration of FASP with protein extraction techniques still requires validation, which typically relies on the assessment of proteome coverage in comparison to other sample preparation pipelines (Wiśniewski, 2019). In these experiments, FASP [which was originally proposed as the method for digestion of cell lysates (Wiśniewski et al., 2009)] was shown to be widely applicable to human and animal samples (Wiśniewski and Mann, 2012). Moreover, this method was successfully employed in proteomics analyses of plant organs—leaves (Wang et al., 2018), seeds (Min et al., 2019), roots (Jiang et al., 2017), and fruits (Szymanski et al., 2017). Thereby, FASP proved to be compatible with all three major protein isolation strategies—phenol extraction, precipitation with TCA/acetone, and their combination (Wang et al., 2018; Heyer et al., 2019).

Although the efficiency of FASP for plant samples was characterized in terms of protein identification rates and sequence coverage, the potential of this technique for quantitative plant proteomics remains completely unknown. Indeed, high contents of proteases, carbohydrates, and secondary metabolites, including protein-binding polyphenols, characteristic for recalcitrant plant tissues, might interfere with protein extraction and MS analysis, dramatically affecting, thereby, the linear dynamic range (LDR) of protein quantification. However, the information about the LDR of protein quantification by FASP-based bottom-up shotgun proteomics is still missing. Moreover, the impact of specific protein isolation techniques in the overall result of such experiments is also unknown. Hence, the FASP-based sample preparation methods still require validation in terms of their applicability for quantitative assessments. Therefore, to fill this gap, we addressed the potential of FASP in combination with two protein isolation protocols for quantification of *Arabidopsis thaliana* leaf and pea (*Pisum sativum* L.) seed proteins by LC-MS-based bottom-up shotgun proteomics. Due to the presence of strongly dominating major proteins (RuBisCO in leaves and storage proteins in seeds), these organs belong to the most difficult plant matrices, i.e., the most strongly affecting protein quantitation. Therefore, we evaluated the LDR of protein quantification in the presence of these complex plant matrices processed by two alternative sample preparation workflows, each in comparison to conventional FASP-based digestion protocols of cell lysates. Our results indicate the applicability of FASP for quantitative plant proteomics with a limited impact of protein isolation technique used on the overall method performance.

## Materials and methods

### Materials and reagents

Materials were obtained from the following manufacturers: Biowest (Nuaillé, France): fetal bovine serum, South America; Capricorn Scientific GmbH (Ebsdorfergrund, Germany): RPMI 1640, Dulbecco’s PBS (1×), penicillin/streptomycin (100×), L-glutamine solution (200 mmol/L), and trypsin-EDTA (0.05%) in DPBS (1×); Carl Roth GmbH and Co (Karlsruhe, Germany): tris(hydroxymethyl)aminomethane (tris, ultra-pure grade), tetramethylethylenediamine (TMED, p.a.), ammonium persulfate (ACS grade), glycerol (p.a.), and bovine serum albumin (BSA); CDS Analytical, LLC (Oxford, PA, USA): Empore Extraction C18 Disks; Honeywell (Charlotte, NC, USA): acetonitrile (LC-MS grade) and methanol (LC-MS grade); PanReac AppliChem (Darmstadt, Germany): glycerol (ACS grade), phenylmethylsulfonyl fluoride (PMSF), and polysorbate 20 (Tween^®^ 20); SERVA Electrophoresis GmbH (Heidelberg, Germany): 2-mercaptoethanol (research grade), acrylamide/bis-acrylamide solution [37.5/1, 30% (*w/v*), 2.6% C], NB sequencing grade modified trypsin from porcine pancreas, and sodium dodecyl sulfate (SDS, electrophoresis grade); Thermo Fisher Scientific (Bremen, Germany): PageRuler™ Prestained Protein Ladder #26616 (10–180 kDa); VWR Chemicals, LLC (Solon, OH, USA): phenol (ultra-pure). Amicon^®^ Ultra-0.5 Centrifugal Filter Unit of 30 kDa molecular weight cutoff (MWCO) and all other chemicals were purchased from Merck KGaA (Darmstadt, Germany).

The prostate cancer cell line PC-3 was obtained from German Collection of Microorganisms and Cell Cultures GmbH and maintained routinely in complete RPMI medium 1640 supplemented with 10% of heat-inactivated FBS, 1% glutamine, and 1% penicillin/streptomycin at 37°C in a humidified atmosphere with 5% CO_2_ to reach subconfluency (~80%) prior to subsequent usage or subculturing (Smolko et al., 2020). For protein isolation, cells were washed three times with ice-cold PBS solution and harvested by adding the detergent-containing extraction buffer.

Pea seeds of the cultivar “Millennium” were obtained from the Research and Practical Center of the National Academy of Science of the Republic of Belarus for Arable Farming (Zhodino, Belarus, harvested in the year 2015 and stored at 4°C). *Arabidopsis thaliana* (Columbia 1092) seeds were planted in wet soil–sand mixture, and the plants were grown in a phytotron MLR-351H (Sanyo Electric Co., Ltd., Moriguchi, Japan) under a short day with an 8-h light (150 ± 2.5 µmol photons m^−2^ s^−1^)/16-h dark cycle at 23/18°C, respectively, and 60% humidity. The plants were harvested after 6 weeks of growth, and the leaves were ground in liquid nitrogen using a Mixer Mill MM 400 ball mill with 3-mm-diameter stainless steel balls (Retsch, Haan, Germany) at a vibration frequency of 30 Hz for 2 min. The plants were characterized elsewhere (Bilova et al., 2016).

### Protein isolation

Protein isolation relied on two approaches: (i) the phenol extraction method described in detail previously (Antonova et al., 2019) and (ii) treatment with an SDS-based solution according to the procedure recently introduced by Bassal et al. (2020) with minor modifications. All experiments were performed in triplicate. In detail, protein isolation from PC-3 cells and a mixture of pea seed powder and Arabidopsis leaf material was accomplished by treatment with extraction buffer [4% (*w/v*) SDS, 10 mmol/L dithiothreitol (DTT), 10 mmol/L EDTA, and 1 mmol/L PMSF in 50 mmol/L Tris-HCl, pH 8.0], followed by two steps of incubation (900 rpm; at 95°C for 10 min, at room temperature for 20 min) and centrifugation (25,000 *g*, 30 min, 10°C) after each incubation. The resulting supernatants were transferred to new tubes.

The total protein fractions from pea seeds and mixtures of pea seed powder and Arabidopsis leaf material were isolated by the phenol extraction procedure described in detail previously (Antonova et al., 2019), and dry protein pellets were reconstituted in 10% (*w/v*) SDS solution. The protein contents were determined by the 2D Quant Kit according to the manufacturer’s instructions.

### FASP protocol

The Amicon^®^ Ultra 30K filter units were conditioned (passivated) on the day prior to digestion with 5% (*v/v*) aqueous Tween^®^ 20 under continuous shaking overnight. Afterwards, filters were washed three times for 10 min with distilled water. The protein aliquots (50 μg) were adjusted to the total volume of 200 μl with urea solution (8 mol/L urea in 50 mmol/L Tris-HCl, pH 8.0), applied to the filter unit and centrifuged (here and below—14,000 *g*, 10 min). The concentrated samples were washed three times with 200 μl of urea solution followed each time by centrifugation. Disulfide bonds were reduced by the addition of 100 μl of a solution of 100 mmol/L DTT and 8 mol/L urea in 50 mmol/L Tris-HCl, pH 8.0, and incubation at 22°C for 1 h under continuous shaking (450 rpm) followed by centrifugation. Alkylation of sulfhydryl groups was accomplished by the addition of 100 µl of 50 mmol/L iodoacetamide in a solution of 8 mol/L urea in 50 mmol/L Tris-HCl, pH 8.0, and incubation at 22°C for 1 h in the dark under continuous shaking followed by centrifugation. The resulting concentrated samples were washed three times with 200 µl of urea solution and three times with 100 μl of 50 mmol/L aq. NH_4_HCO_3_ followed each time by centrifugation. Afterwards, the proteins were digested by sequential addition of two aliquots of 2.5 and 1 μg trypsin, reconstituted in 50 μl of 50 mmol/L aq. NH_4_HCO_3_ (enzyme-to-protein ratio 1:20 and 1:50 *w/w*, respectively) and incubation at 37°C for 4 and 12 h under continuous shaking (450 rpm), respectively. The digests were collected by centrifugation, and the filter units were rinsed with 40 μl of 50 mmol/L aq. NH_4_HCO_3_ three times followed by centrifugation. The resulting filtrates were desalted by solid phase extraction (SPE) as described by Mamontova et al. (2019). The completeness of tryptic digestion was verified by SDS-PAGE as described by Greifenhagen et al. (2016).

### LC-MS/MS

All samples were analyzed by nanoRP-HPLC-ESI-MS/MS using an Orbitrap XL hybrid mass spectrometer equipped with a NanoFlex source, coupled online to an Ultimate 3000 nano-HPLC system (Thermo Fisher Scientific, Bremen, Germany). Proteolytic peptides (1 μg) were loaded on a trap column (PepMap 100 C18, 300 μm × 5 mm, particle size 5 μm) during 15 min with 0.1% (*v/v*) TFA at a flow rate of 30 μl/min and resolved on a separation column (PepMap 100 C18, 75 μm × 150 mm, particle size 3 μm, Thermo Fisher Scientific, Bremen, Germany). The peptides were separated with the linear gradient from 3% to 35% eluent B over 90 min (A: 0.1% *v/v* aqueous formic acid, B: 0.08% *v/v* formic acid in acetonitrile) at a flow rate of 300 nl/min. The raw files were acquired as data-dependent acquisition (DDA) experiments accomplished in positive ion mode. Dependent tandem mass spectrometric (MS/MS) experiments relied on higher-energy collision-induced dissociation (HCD) at 27% normalized collision energy (NCE). MS data (*m/z* range 300–1500) were recorded with *R* = 60,000, the target of the automated gain control (AGC) was set to 2 × 10^5^, and the maximum injection time was 50 ms. Each full scan was followed by high-resolution HCD product ion scans within 5 s, starting with the most intense signal in the mass spectrum, with charge states ranging from 2 to 6. For MS/MS scans, the following parameters were applied: a resolution (*R*) of 15,000, an AGC of 5 × 10^4^, and a maximum injection time of 200 ms. Dynamic exclusion of multi-charged peptide ions was set to 60 s. Targeted analyses relied on a nonscheduled SRM acquisition method to quantitate up to 10 peptides in one run (three transitions per peptide). Mass tolerances for searching precursor and fragment ions (defined as trap isolation window) were ± 0.5 and 1.5 Da, respectively. The mass spectrometry proteomics data were deposited to the ProteomeXchange Consortium *via* the PRIDE (Perez-Riverol et al., 2019) partner repository with the dataset identifier PXD025897 and 10.6019/PXD025897.

### Data analysis

Identification of peptides relied on Proteome Discoverer software (version 2.2.0.388, Thermo Fisher Scientific, Bremen, Germany) and Sequest HT search engine. For database search, the following UniProt reference FASTA files were used: *A. thaliana* (39,299 entries, downloaded 7 September 2019) and BSA (entry P02769, downloaded 29 October 2019). The enzyme was set to trypsin, tolerating two missed cleavages. The precursor and fragment mass tolerance were set to 10 ppm and 0.8 Da, respectively. Carbamidomethylation of cysteine was employed as a fixed modification, and oxidation of methionine was specified as a variable one. False discovery rate (FDR) was set to 0.05. Proteotypic peptides were selected for integration if they were confidently (XCorr ≥ 2.20) annotated as [M+2H]^2+^ ions, and did not contain missed cleavage sites, modifications, and methionine or cysteine amino acid residues. The peak integration was accomplished in the Quan Browser application of the Xcalibur software (Thermo Fisher Scientific). Detection and integration of the peptide-specific peaks in corresponding extracted ion chromatograms (XICs, *m/z* ± 0.02) were accomplished by ICIS algorithm with the following parameters: Smoothing Points 15, Baseline Window 40–80, Area Noise Factor 5, and Peak Noise Factor 10. The linearity of protein quantification was assessed for several proteotypic peptides by Xcalibur software (version 2.0.7, Thermo Fisher Scientific, Bremen, Germany). For relative quantification of SRM data, the peak areas were determined using Skyline software (version 22.2.0.255, MacCoss Lab Software, USA, https://skyline.ms/project/home/software/Skyline/begin.view). The default transition settings were applied except for method match tolerance *m/z* that was set to 0.6. Intensity of each proteotypic peptide was calculated as a sum of chromatographic area of each fragment ion.

## Results and discussion

### Quantitative analysis of BSA spiked in cell lysates

Originally, FASP was proposed for (human/animal) cell lysates (Wiśniewski et al., 2009), and its performance was comprehensively characterized with various cell lines and experimental setups (Wiśniewski et al., 2009; Ni et al., 2017; Potriquet et al., 2017). Therefore, here we decided for cultured cells as a reference to estimate the linear performance of this method. Following the previously established methodology, prostate cancer (PC-3) cells were lysed with extraction buffer containing 4% (*w/v*) SDS, incubated at 95°C, and centrifuged; i.e., the procedure reproduced the classical workflow of Wiśniewski et al. (2009). Of course, the total cell lysate represents a complex system, highly prone to matrix effects (most probably, and predominantly, ion suppression), (Mitchell, 2010), which can affect the LDRs of individual tryptic peptides.

To get access to the LDRs in the easiest and most straightforward way, we applied a spiking approach, which represents a well-established normalization strategy in label-free quantification (LFQ) experiments (Tuli et al., 2012). Specifically, after determination of protein concentrations, aliquots of cell lysates were spiked with BSA at the percentage concentration ratios of 3.125%, 6.25%, 12.5%, 25%, 50%, and 100% (*w/w*) that corresponded to 0.47, 0.94, 1.88, 3.76, 7.5, and 15 pmol of BSA loads, respectively. This range of ratios covered two orders of magnitude that in most of the cases is sufficient for characterization of typical protein expression responses, i.e., allows monitoring up to 100-fold alteration in expression of individual proteins. Thereby, based on earlier performed linearity tests, the point 15 pmol is above the LDR (Zauber et al., 2013). As the protein expression dynamic range in both cell lysates and plant protein extracts accounts at least seven orders of magnitude (Geiger et al., 2012), specific groups of individual proteins can be addressed by optimization of the protein extraction scale and by a broad selection of enrichment, depletion, and fractionation methods (Ly and Wasinger, 2011).

Although spiking with mixtures of standard peptides represents an adequate approach to estimate method linearity performance (Beri et al., 2015), we decided here for the spike with allogenic protein, as this approach might consider not only matrix effects, but also the contribution of factors related to proteolysis. Bovine serum albumin seems to be a suitable protein for this purpose. Indeed, it has 58 lysyl and 24 arginyl residues, which are relatively homogeneously distributed in the protein sequence. It gives a rich selection of potential proteotypic peptides covering the whole range of peptide-specific retention times. Indeed, serum albumin was established as a tool for reference protein normalization in both cell (Chang et al., 2012) and plant (Zauber et al., 2013) proteomics. Thus, the behavior of its proteolytic peptides in plant protein hydrolysates is well-characterized and is in agreement with dynamic ranges of cell- and plant-derived tryptic peptides. This, in turn, will ensure comparability of the results obtained with cell lysates and plant protein extracts. In agreement with earlier reports (Ni et al., 2017; Potriquet et al., 2017), FASP proved to be an efficient tool for the analysis of cell lysates. The completeness of tryptic digestion was verified by SDS-PAGE and also was in agreement with the results of previous studies (Wiśniewski et al., 2009).

**Figure 1:**
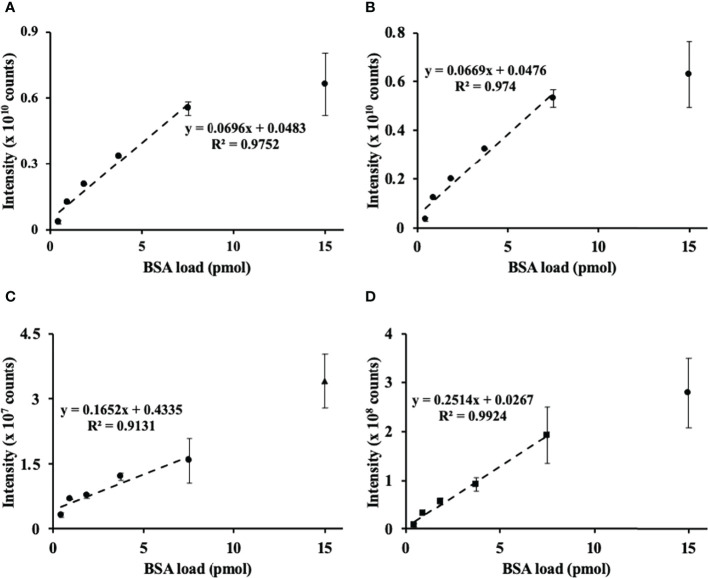
Assessment of method linearity for the quantification of bovine serum albumin (BSA) spiked to the prostate cancer (PC-3) cell lysate to obtain 0.47, 0.94, 1.88, 3.76, 7.5, and 15 pmol of BSA loads. Quantification of BSA relied on the sum **(A)** of integrated and individual peak areas obtained for *m/z* 582.3190 ± 0.02, 464.2504 ± 0.02, and 756.4250 ± 0.02 at t_R_ 78.4, 65.9, and 75.9, corresponding to the [M+2H]^2+^ ions of the proteotypic tryptic peptides LVNELTEFAK **(B)**, YLYEIAR **(C)**, and VPQVSTPTLVEVSR **(D)**, respectively. The peak integration was accomplished in the Quan Browser application of the Xcalibur software (Thermo Fisher Scientific).

After the nanoRP-HPLC-ESI-LIT-Orbitrap-MS analysis, the raw files were searched by SEQUEST engine against the BSA sequence database. Sequence coverage of BSA spiked to cell lysates corresponded to 83% with 84 identified unique peptides (for the sequences, see Supplementary information 2, **Tables S2-1**). Based on this search, three proteotypic BSA peptides, namely, LVNELTEFAK, AEFVEVTK, and YLYEIAR, with *m/z* 582.3190 ± 0.02, 464.2504 ± 0.02, and 756.4250 ± 0.02 at t_R_ 52.4, 45.6, and 50.2, corresponding to the [M+2H]^2+^ ions, respectively, were used to assess the linearity of the sample preparation method. The method delivered acceptable linear correlation (*R* ^2^ = 0.975) over the half of the assessed dynamic range (**Figure 1A**) based on the relative intensity of the overall BSA abundance response (calculated as an integrated sum of corresponding peptide signal intensities) and its contents spiked to aliquots of cell lysates. Accordingly, each of the individual peptides demonstrated an excellent linearity of response *R* ^2^ from 0.91 to 0.99 (**Figures 1B–D**).

Thus, in our hands, the FASP-based quantitation of BSA spiked to the cell lysate proved to be reliable at the low pmol levels. This result was in agreement with the classical works published before (Wiśniewski, 2019). Based on this finding, we assume that the PC-3 cell lysate spiked with BSA is an appropriate reference for our plant shotgun proteomics experiments.

### Quantitative analysis of BSA spiked to the protein extracts of the pea seeds

Having the verified FASP method in hand, we extended our spike approach to pea seed total protein fraction. For this aim, proteins were isolated from the plant tissues by phenol extraction, dry protein pellets were reconstituted in 10% (*w/v*) SDS solution, and protein concentrations were determined. Based on these values, a 1 g/L BSA solution in 10% SDS was spiked to protein solution aliquots using the scheme described above for the cell lysates. Thus, the digestion protocol of Mann’s group was transferred to the appropriate protein extraction method, which is currently considered as the most efficient one in terms of sample quality and protein identification rates (Saravanan and Rose, 2004; Carpentier et al., 2005).

**Figure 2:**
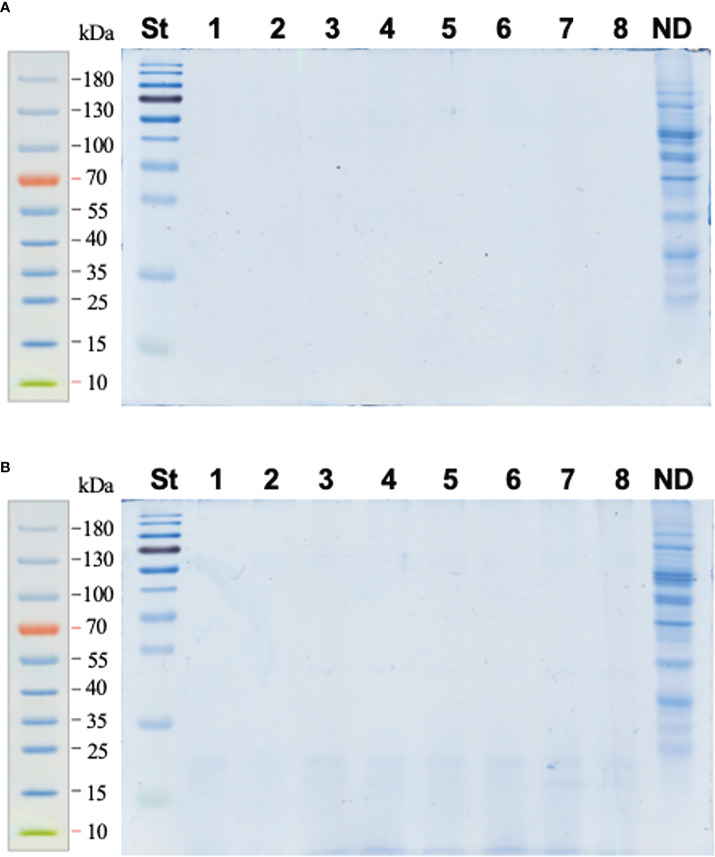
SDS-PAGE electrophoreograms acquired for the pea seed protein digested with trypsin using the FASP approach (a total of 50 μg of protein was applied to each filter unit). Lines 1–8 correspond to samples of digested pea seed protein with the spiked BSA at the percentage concentration ratios of 12.5 (bands 1–3), 6.25 (bands 4–6), and 3.125 (bands 7–8). The analyses were performed with filtrate **(A)** and the fraction retained on the filter **(B)** after 3× washing with 40 μl of 50 mmol/L aq. NH_4_HCO_3_ and centrifugal filtration (14,000 *g*, 10 min). To assess the completeness of hydrolysis, 5 µg of each digest was applied on the gel. The whole retained fraction (corresponding to 50 µg of digested protein) was completely transferred to a polypropylene tube, lyophilized, reconstituted in SDS-PAGE sample buffer, and loaded on the gel. The overall lane densities were compared to those of non-digested (ND) protein (5 μg) applied to a separate lane. St – Page Ruler Prestained Protein Ladder.

The FASP approach proved to be an appropriate method for digestion of the pea seed proteins; i.e., it was ideally compatible with the phenol extraction method. Indeed, most of the total seed protein fraction (97.7%) was successfully digested and transferred through the cellulose membrane of the filter units (**Figure 2A**), whereas only 2.3% of the protein aliquot, subjected to proteolysis, remained on the membrane after 3× washing with 50 mmol/L aq. NH_4_HCO_3_ (as the averaged total lane abundance of the non-filtered fraction constituted 23% of the ND reference, which, in turn, corresponded to 10% of the digested aliquot, **Figure 2B**). These assessments relied on our quantitative approach, assuming sensitivity of colloidal Coomassie of about 30 ng/band (Frolov et al., 2014).

**Figure 3:**
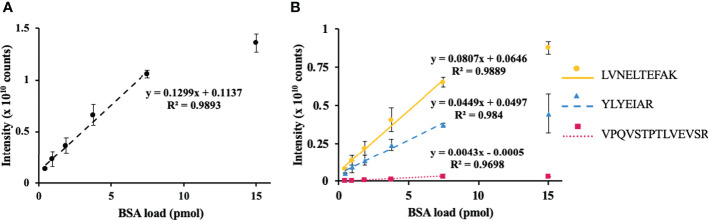
Assessment of method linearity for the quantification of bovine serum albumin (BSA) spiked to the total pea seed protein to obtain 0.47, 0.94, 1.88, 3.76, 7.5, and 15 pmol of BSA loads. Seed proteins were isolated by phenol extraction. Quantification of BSA relied on the sum **(A)** of integrated peak areas obtained for *m/z* 582.3190 ± 0.02, 464.2504 ± 0.02, and 756.4250 ± 0.02 at t_R_ 78.1, 66.0, and 75.7, corresponding to the [M+2H]^2+^ ions of the proteotypic tryptic peptides LVNELTEFAK (circles, solid line), YLYEIAR (triangles, dashed line), and VPQVSTPTLVEVSR (squares, dotted line), respectively. Quantification of individual peptides is shown on panel **(B)** The peak integration was accomplished in the Quan Browser application of the Xcalibur software (Thermo Fisher Scientific).

To address the efficiency of protein quantification with the FASP approach, the integrated peak areas were calculated for each peptide signal in corresponding extracted ion chromatograms (XICs, *m/z* ± 0.02) and summed for each concentration point. This approach yielded the same LDR as was observed above for the PC-3 cells (**Figure 3A**). For all three selected proteotypic BSA peptides, similar concentration–signal intensity curves were observed (**Figure 3B**). Thereby, the response was linear for up to 50% of spiked BSA (*R* ^2^ = 0.97–0.99).

As the next logical step, implementation of the selected reaction monitoring (SRM) as a quantitation method allowed extending the LDR of our approach down to the femtomole level. For this aim, the pea seed proteins were spiked with BSA amounts corresponding to 1, 4, 16, 62.5, 125, 500, and 2000 fmol of BSA column loads (i.e., we extended the dynamic range of our experiment three orders of magnitude down). The quantification relied on five proteotypic BSA peptides (7–20 residues, not containing methionine and internal trypsin cleavage sites) and the SRM acquisition method with three specific combinations of precursor and product *m/z* ranges per peptide (transitions) yielding the highest signal intensity in the MS/MS spectra of the DDA experiment (**Table S1-1**). Quantitative analysis was performed using Skyline software (**Figure S1-1**). The analysis yielded limits of detection (LODs) for the peptides DAFLGSFLYEYSR and LVNELTEFAK as low as 4 fmol and limits of quantification (LOQs) of 125 fmol (**Table S1-2**; **Figures S1-2A, B**), which corresponded to LDRs of 0.6 × 10^2^. These values corresponded well to the published data for LTQ instruments and allowed comparable sensitivity of BSA quantification (Ishihama et al., 2005). The other selected proteotypic peptides obviously had lower ionization efficiency and showed, therefore, less favorable LODs and LOQs (**Table S1-2** and **Figures S1-2C–E**).

This result highlights the general applicability of FASP in quantitative plant proteomics, i.e., its combination with phenol extraction gave access to acceptable linearity for quantification of BSA spiked to plant protein isolates (**Figure 3**). Moreover, BSA spiked to pea seed protein yielded superior sequence coverage (92%) and a higher number of identified unique peptides (93) in comparison to BSA spiked to PC-3 cell lysate. This fact can be explained by the higher efficiency of phenol extraction with respect to discrimination from non-protein contaminations (Isaacson et al., 2006). On the other hand, this observation can be attributed to different relative abundances of individual proteins in these matrices. Indeed, pea seeds contain several strongly dominant proteins; most proteins are much less abundant (Mamontova et al., 2018). This might result in lower ion suppression at most of the chromatogram span.

### Quantitative analysis of Arabidopsis proteins in pea seed protein matrix

At the next step, we addressed the impact of the protein isolation method on the performance of FASP-based quantitative proteome analysis. To get access to this information, we compared the linearity of two protein isolation methods: (i) phenol extraction and (ii) treatment with a detergent-containing solution followed by incubation at 95°C. For this purpose, to simulate different relative representation of proteins in the proteome, frozen milled Arabidopsis leaf material was added to the pea seed powder at the percentage concentration ratios 10%, 25%, 50%, 75%, 90%, and 100% (*w/w*). The total protein of the resulting mixtures was co-extracted using the phenol extraction procedure or treatment with SDS-containing extraction solution. The completeness of tryptic digestion was verified by SDS-PAGE (**Figure S1-3**).

As can be seen from **Table S1-3**, the efficiency of the two applied digestion protocols clearly differed—the detergent-based protocol yielded 11% more identified peptides, 17% more possible proteins, and 24% more identified non-redundant proteins in comparison to the phenol extraction. However, the phenol-based protocol yielded 12% more identified membrane protein than the former procedure (1,078 vs. 945, **Table S1-4**), although it was less specific for trans-membrane domains (**Table S1-5**). For the complete lists of the Arabidopsis proteins identified by both isolation protocols, see **Tables S2-2** and **S2-3**.

**Figure 4:**
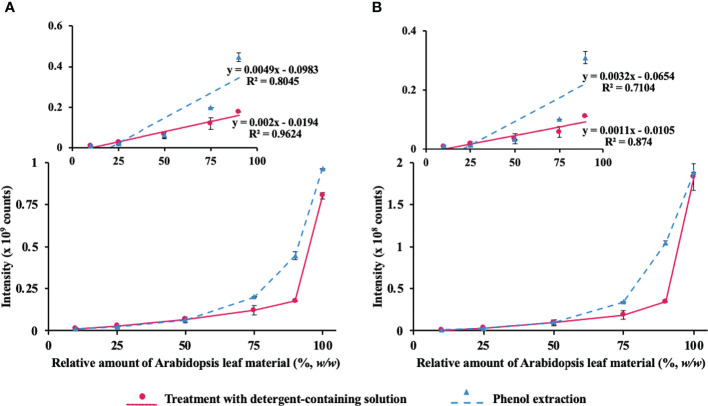
Assessment of the method linearity for quantification of *Arabidopsis thaliana* leaf proteins ribulose-1,5-bisphosphate carboxylase-oxygenase (RuBisCO) and RuBisCO activase after co-extraction from Arabidopsis leaf material added to pea seed powder at the different percentage concentrations [10%, 25%, 50%, 75%, 90%, and 100% (*w/w*)] using the phenol extraction procedure (triangles, dashed line) or treatment with SDS-containing extraction solution (circles, solid line). Quantification of RuBisCO **(A)** relied on the sum of integrated peak areas obtained for *m/z* 511.2693 ± 0.02, 614.8302 ± 0.02, and 704.3376 ± 0.02 at t_R_ 60.0, 49.4, and 44.6, corresponding to the [M+2H]^2+^ ions of the proteotypic tryptic peptides DTDILAAFR, DLAVEGNEIIR, and LTYYTPEYETK, respectively, whereas RuBisCO activase **(B)** was quantified with *m/z* 504.2741 ± 0.02, 849.3843 ± 0.02, and 576.8606 ± 0.02 at t_R_ 42.2, 40.9, and 67.9, corresponding to the [M+2H]^2+^ ions of the proteotypic tryptic peptides FVESLGVEK, GLAYDTSDDQQDITR, and VPLILGIWGGK, respectively. The peak integration was accomplished in the Quan Browser application of the Xcalibur software (Thermo Fisher Scientific).

As protein yields from pea seeds were eightfold higher in comparison to Arabidopsis leaf (5.4 vs. 42.2 mg/g fresh weight), even the most abundant Arabidopsis proteins acted as minor components of the mixed protein isolates. Therefore, we followed the abundance of two proteins, characteristic for Arabidopsis leaves—large subunit of chloroplastic ribulose bisphosphate carboxylase/oxygenase (RuBisCO, the most abundant leaf polypeptide, **Figure 4A**) and RuBisCO activase, which is less abundant (**Figure 4B**). The first protein was quantified with *m/z* 511.2693 ± 0.02, 614.8302 ± 0.02, and 704.3376 ± 0.02 at t_R_ 60.0, 49.4, and 44.6, respectively. These *m/z* values corresponded to [M+H]^2+^ ions of proteotypic peptides DTDILAAFR, DLAVEGNEIIR, and LTYYTPEYETK, respectively, whereas the signals at *m/z* 504.2741 ± 0.02, 849.3843 ± 0.02, and 576.8606 ± 0.02 with t_R_ 42.2, 40.9, and 67.9, corresponded to the [M+2H]^2+^ ions of the proteotypic tryptic peptides FVESLGVEK, GLAYDTSDDQQDITR, and VPLILGIWGGK, respectively.

As can be seen from **Figure 4** (and its more detailed presentation in **Figure S1-4**), direct extraction with SDS-containing solution provided better linearity (higher maximal point of the LDR) of the integrated response of Arabidopsis proteins, each based on the sum of the selected three proteotypic peptides. Furthermore, we implemented SRM-MS for deeper investigation of the linearity behavior of Arabidopsis proteins. The quantification relied on 20 proteotypic peptides (7–20 residues, not containing methionine and internal trypsin cleavage sites) representing six proteins of different abundance and SRM acquisition method with three specific combinations of precursor and product *m/z* ranges per peptide (transitions) yielding the highest signal intensity in the MS/MS spectra of the DDA experiment (**Table S1-6**). Quantitative analysis was performed using Skyline software (**Figure S1-5**). Based on the obtained data, linear regression curves could be built and the sensitivity and linearity parameters could be assessed (**Figures S1-6** and **S1-7**). The analysis yielded the LODs and LOQ at the level of 10% of Arabidopsis material for peptides DTDILAAFR and ESTLGFVDLLR of RuBisCO large chain, as well as for VPLILGIWGGK and NILLNEGIR of RuBisCO activase and Photosystem II D2 protein, respectively. The other selected proteotypic peptides obviously had lower ionization efficiency and showed, therefore, less favorable LODs and LOQs (**Tables S1-7** and **S1-8**). For all analyzed proteins, the detergent-based extraction showed compromised (in comparison to the phenol extraction) recovery of the corresponding proteins in SDS-containing extraction buffer when Arabidopsis leaf material contributed more than 50% in the plant material mix (**Figures S1**–**4**). Indeed, when the detergent solution was used for extraction, linearity was superior (*R* ^2^ values better than 0.95) up to a contribution of Arabidopsis material accounting 90%, whereas for phenol extraction, *R* ^2^ values typically were 0.85 or lower. However, when the quantification dynamics range was reduced to a contribution of Arabidopsis material accounting 50%, both detergent and phenol extraction methods yielded similar linearity (*R* ^2^ values of 0.99 and 0.97, respectively, data not shown).

Most likely, the observed differences were attributed to stronger matrix effects, which might accompany detergent-based extraction. Indeed, although all reagents used in the digestion are quantitatively removed during sample preparation and by online trapping in terms of the nano-LC setup, secondary metabolites (which are co-extracted with proteins in this design) cannot be quantitatively removed by both these steps and might cause inhibition of trypsin activity and ion suppression *via* co-elution with individual peptides in RP-HPLC experiments (Wang et al., 2018). On the other hand, phenol isolates are free from non-protein contaminants, and pea proteolytic peptides represented the only factor of ion suppression in our experimental system. Accordingly, the signal intensity of Arabidopsis tryptic peptides increased when the contribution of pea seed protein in the total isolate decreased.

It is worth noting that the presented approach has limitations. The spike-in experiments are quite convenient and, certainly, suitable for validation of label-free proteomics protocol, as the differences between samples are known, and method performance can be reliably characterized by the ability to identify the true differences (Riter et al., 2011). However, testing the protocol in a case study representing typical real research is another important part of the validation procedure (Riter et al., 2011; Välikangas et al., 2018) and needs to be its next step. Indeed, even though the presented FASP methodology proved to be efficient in the spike-in experiments, its performance in identifying the biologically relevant alterations in protein dynamics can be different.

Moreover, our experimental setup relied on the whole plant organs, whereas currently single-cell proteomics becomes the main road of the state-of-the-art proteomics, as it gives much better insight into biological processes without averaging effects (Kelly, 2020). However, unfortunately, plant systems bring unique challenges for single-omics experiments such as optimization of individual cell isolation from different plant species and plant organs, determining and detecting cell type-specific marker genes as well as data analysis methods (Clark et al., 2022). For this reason, the protocol validation for whole organ lysates represents a critical, absolutely mandatory, and not avoidable step in obtaining biologically relevant information.

Thus, protein isolation protocol affects the LDR of FASP-based quantitative proteomics techniques. However, this difference in LDRs can be considered during data interpretation and appropriately corrected by experimental design.

## Conclusions

FASP represents a powerful and versatile technique to access quantitative and reproducible protein solubilization and digestion for shotgun proteomics. It was originally proposed for cell lysates in the mid-2000s by Mann’s group. Since that time, it was comprehensively optimized and validated for cells, blood plasma, and homogenates of animal organs. Finally, during recent years, FASP was employed in plant proteomics as well. However, in our opinion, this step requires a comprehensive estimation of the methods’ behavior with respect to plant matrix, which is known to be a much more complex biological material that is difficult to handle when compared to mammalian cells. The most critical aspect here is the effect of the plant matrix on LDRs of individual proteins. This knowledge is critically important for a correct assessment of quantitative alterations. Here, to the best of our knowledge, for the first time, we provide data on the linearity of FASP in plant matrix. Surprisingly, when coupled to plant-specific protein isolation protocols, this method demonstrates even better performance in comparison to mammalian matrices. The selection of the protein isolation protocol for plant FASP assumes a compromise between recovery (which is more favored by the phenol extraction method) and LDR (which is better when direct detergent treatment is applied). Therefore, a linearity/recovery test prior to working with a new plant matrix is mandatory for obtaining adequate quantitative information.

## Data availability statement

The datasets presented in this study can be found in online repositories. The names of the repository/repositories and accession number(s) can be found below: http://www.proteomexchange.org/, PXD025897.

## Author contributions

TL performed protein isolation, FASP of pea seed and Arabidopsis leaf proteins, and data analysis and wrote the manuscript draft. CI performed proteomics analysis. MS performed FASP of cell proteins. RR supervised cell experiment and contributed to the writing of the final draft of the manuscript. LW contributed to the writing of the final draft of the manuscript. AF proposed the idea of the manuscript, supervised the work, and wrote the final draft of the manuscript. All authors contributed to the article and approved the submitted version.

## Funding

The financial support from the Russian Foundation of Basic Research (#20-54-00044 and #20-34-90160), Deutsche Forschungsgemeinschaft (DFG, project #FR3117/2-3), and Leibniz Society is gratefully acknowledged.

## Acknowledgments

We thank Dr. Wolfgang Höhenwarter for fruitful discussions.

## Conflict of interest

The authors declare that the research was conducted in the absence of any commercial or financial relationships that could be construed as a potential conflict of interest.

## Publisher’s note

All claims expressed in this article are solely those of the authors and do not necessarily represent those of their affiliated organizations, or those of the publisher, the editors and the reviewers. Any product that may be evaluated in this article, or claim that may be made by its manufacturer, is not guaranteed or endorsed by the publisher.
