# Supplementary material for: Does filter-aided sample preparation provide sufficient method linearity for quantitative plant shotgun proteomics?
Source: Front Plant Sci. 2022 Nov 23;13:874761. doi: 10.3389/fpls.2022.874761 (PMC9728026; doi:10.3389/fpls.2022.874761)
Supplement: Supplementary file 1 [file DataSheet_1.pdf]

Does filter aided sample preparation (FASP) provide sufficient method linearity for quantitative plant shotgun proteomics?

Tatiana Leonova,[1,2] Christian Ihling,[3] Mohamad Saoud,[1] Nadezhda Frolova,[1,2] Robert Rennert,[1] Ludger A. Wessjohann,[1] Andrej Frolov [1,2*]

Supplementary information 1

[1]Leibniz Institute of Plant Biochemistry, Department of Bioorganic Chemistry, [2]St. Petersburg State University, [3]Martin-Luther Universität Halle-Wittenberg, Institute of Pharmacy, Department of Pharmaceutical Chemistry and Bioanalytics

*Corresponding author:

Dr. Andrej Frolov

Leibniz Institute of Plant Biochemistry

Department of Bioorganic Chemistry

Weinberg 3

06120 Halle (Saale), Germany

Tel. +49(345) 55821350

Fax. +49(345) 55821309

Email: afrolov@ipb-halle.de

Directory

Figure S1-1 Overlays of the extracted ion chromatograms (XICs) obtained for individual fragment ions (specified in the top of each panel) of BSA proteotypic peptides selected for targeted quantification of BSA in the tryptic hydrolysates of the pea seed protein extracts spiked with different amounts of BSA by selected reaction monitoring (SRM): LVNELTEFAK (A), DAFLGSFLYEYSR (B), YLYEIAR (C), YNGVFQECCQAEDK (D), YICDNQDTISSKGWGPDYPR (E). Chromatograms were visualized with Skyline 22.2.0.225 software. 4

Figure S1-2 Linear regression curves obtained for individual BSA tryptic peptides selected for targeted quantification of BSA in the tryptic hydrolysates of the pea seed protein extracts spiked with different amounts of BSA by selected reaction monitoring (SRM): LVNELTEFAK (A), DAFLGSFLYEYSR (B), YLYEIAR (C), YNGVFQECCQAEDK (D), YICDNQDTISSKGWGPDYPR (E). 5

Figure S1-3 SDS-PAGE electrophoreograms acquired for the co-extracted Arabidopsis leaf and pea seed proteins digested with trypsin using the FASP approach (in total 50 µg of protein were applied to each filter unit). 6

Figure S1-4 Assessment of the method linearity for quantification of Arabidopsis leaf proteins ribulose-1,5-bisphosphate carboxylase-oxygenase (RuBisCO, A-C) and RuBisCO activase (D-F) after co-extraction from Arabidopsis leaf material added to pea seed powder at the different percentage concentration (10, 25, 50, 75, 90, 100% (*w/w*)) using the phenol extraction procedure or treatment with SDS-containing extraction solution....................8

Figure S1-5 Overlays of the extracted ion chromatograms (XICs) obtained for individual fragment ions (specified in the top of each panel) of proteotypic peptides selected for targeted SRM quantification of RuBisCO large chain (LTYYTPEYETK, **A**, DTDILAAFR, **B**, ESTLGFVDLLR, **C**), chloroplastic RuBisCO activase (VPLILGIWGGK, **D**, GLAYDTSDDQQDITR, **E**, FVESLGVEK, **F**), cytochrome f (YSEITFPILAPDPATNK, **G**, GLELLVSEGESIK, **H**), myrosinase 1 (GFIFGVASSAYQVEGGR, **I**, YYNGLIDGLVAK, **J**, LPEFSETEAALVK, **K**, NWITINQLYTVPTR, **L**), photosystem II D2 protein (NILLNEGIR, **M**, AAEDPEFETFYTK, **N**, AYDFVSQEIR, **O**), chloroplastic protein PLASTID TRANSCRIPTIONALLY ACTIVE 16 (SQPLTISDLIEK, **P**). Chromatograms were visualized with Skyline 22.2.0.225 software. 11

Figure S1-6 Linear regression curves obtained for individual RuBisCO large chain (DTDILAAFR, **A**, LTYYTPEYETK, **B**, ESTLGFVDLLR, **C**), chloroplastic RuBisCO activase (VPLILGIWGGK, **D**, FVESLGVEK, **E**), photosystem II D2 protein (NILLNEGIR, **F**, AAEDPEFETFYTK, **G**, AYDFVSQEIR, **H**), cytochrome f (YSEITFPILAPDPATNK, **I**), myrosinase 1 (GFIFGVASSAYQVEGGR, **J**, LPEFSETEAALVK, **K**), chloroplastic protein PLASTID TRANSCRIPTIONALLY ACTIVE 16 (SQPLTISDLIEK, **L**) tryptic peptides selected for targeted SRM-based quantification of these proteins in the tryptic hydrolysates obtained after the co-extraction of the mixture of Arabidopsis leaf and pea (*P. sativum*) seed material and co-digestion of the resulted protein isolate by the detergent-based....................13

Figure S1-7 Linear regression curves obtained for individual RuBisCO large chain (DTDILAAFR, **A**, LTYYTPEYETK, **B**, ESTLGFVDLLR, **C**), chloroplastic RuBisCO activase (VPLILGIWGGK,

D, FVESLGVEK, **E**), photosystem II D2 protein (NILLNEGIR, **F**, AAEDPEFETFYTK, **G**, AYDFVSQEIR, **H**), cytochrome f (YSEITFPILAPDPATNK, **I**), myrosinase 1 (GFIFGVASSAYQVEGGR, **J**, LPEFSETEAALVK, **K**), chloroplastic protein PLASTID TRANSCRIPTIONALLY ACTIVE 16 (SQPLTISDLIEK, **L**) tryptic peptides selected for targeted SRM-based quantification of these proteins in the tryptic hydrolysates obtained after the co-extraction of the mixture of Arabidopsis leaf and pea (*P. sativum*) seed material and co-digestion of the resulted protein isolate by the phenol-based....15

Table S1-1 Proteotypic BSA tryptic peptides selected for targeted quantification of BSA by selected reaction monitoring in the tryptic hydrolyzates of the pea seed protein extracts spiked with different amounts of BSA 16

Table S1-2 Sensitivity and linearity data acquired for individual BSA tryptic peptides in the spike with pea seed protein hydrolyzate employed in the targeted quantification experiments with spiked pea seed protein 17

Table S1-3 Number of protein groups, proteins, and individual peptides of *Arabidopsis thaliana* identified after two protein isolation protocols....18

Table S1-4 Number of membrane proteins of *Arabidopsis thaliana* analyzed after two protein isolation protocols 19

Table S1-5 Number of protein regions of Arabidopsis thaliana analyzed after two protein isolation protocols 20

Table S1-6 Proteotypic tryptic peptides of Arabidopsis proteins selected for targeted quantification by selected reaction monitoring in the samples co-extracted and co-digested with pea seed proteins 21

Table S1-7 Sensitivity and linearity data acquired for individual tryptic peptides of Arabidopsis proteins employed in the targeted quantification experiments in the samples co-extracted and co-digested (by the detergent-based protocol) with pea seed proteins 23

Table S1-8 Sensitivity and linearity data acquired for individual tryptic peptides of Arabidopsis proteins employed in the targeted quantification experiments in the samples co-extracted and co-digested (by the phenol extraction protocol) with pea seed proteins 25

Figures

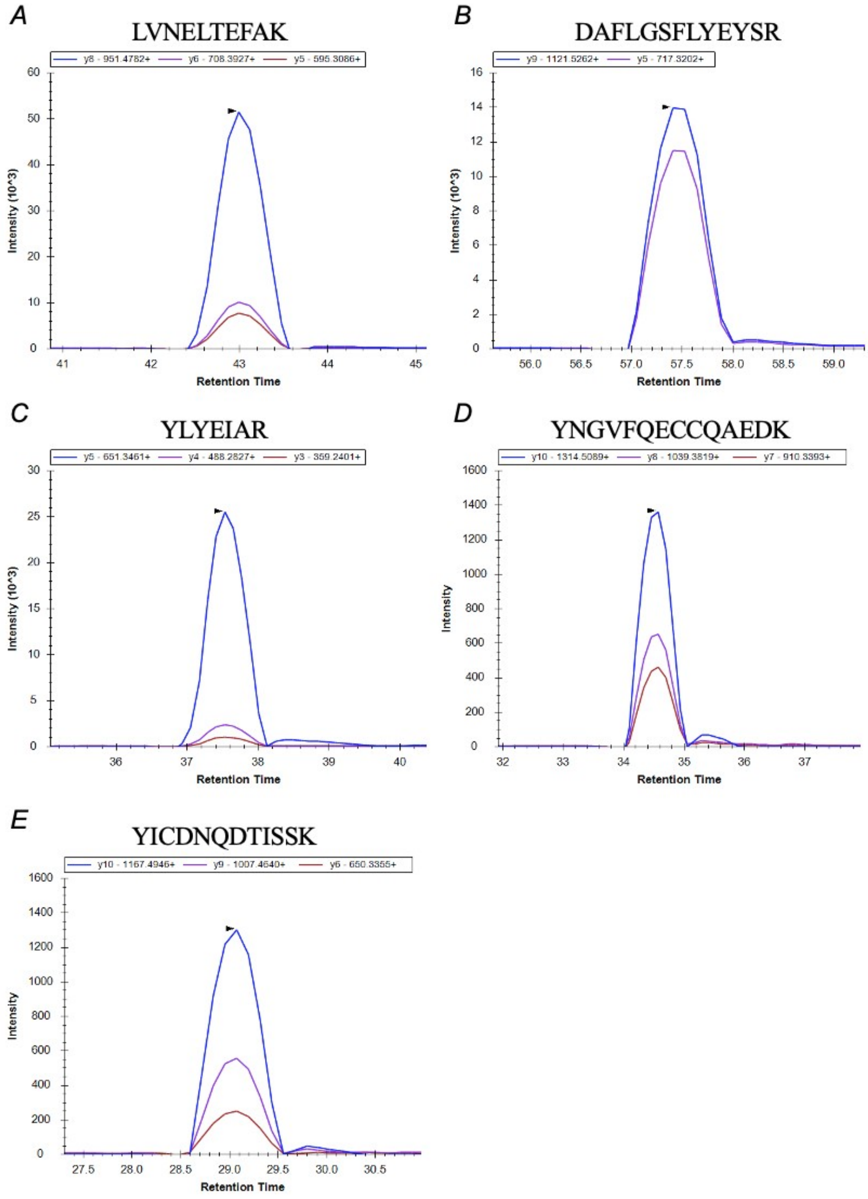

Figure S1-1 Overlays of the extracted ion chromatograms (XICs) obtained for individual fragment ions (specified in the top of each panel) of BSA proteotypic peptides selected for targeted quantification of BSA in the tryptic hydrolysates of the pea seed protein extracts spiked with different amounts of BSA by selected reaction monitoring (SRM): LVNELTEFAK (A), DAFLGSFLYEYSR (B), YLYEIAR (C), YNGVFQECCQAEDK (D), YICDNQDTISSKGWGPDYPR (E). Chromatograms were visualized with Skyline 22.2.0.225 software.

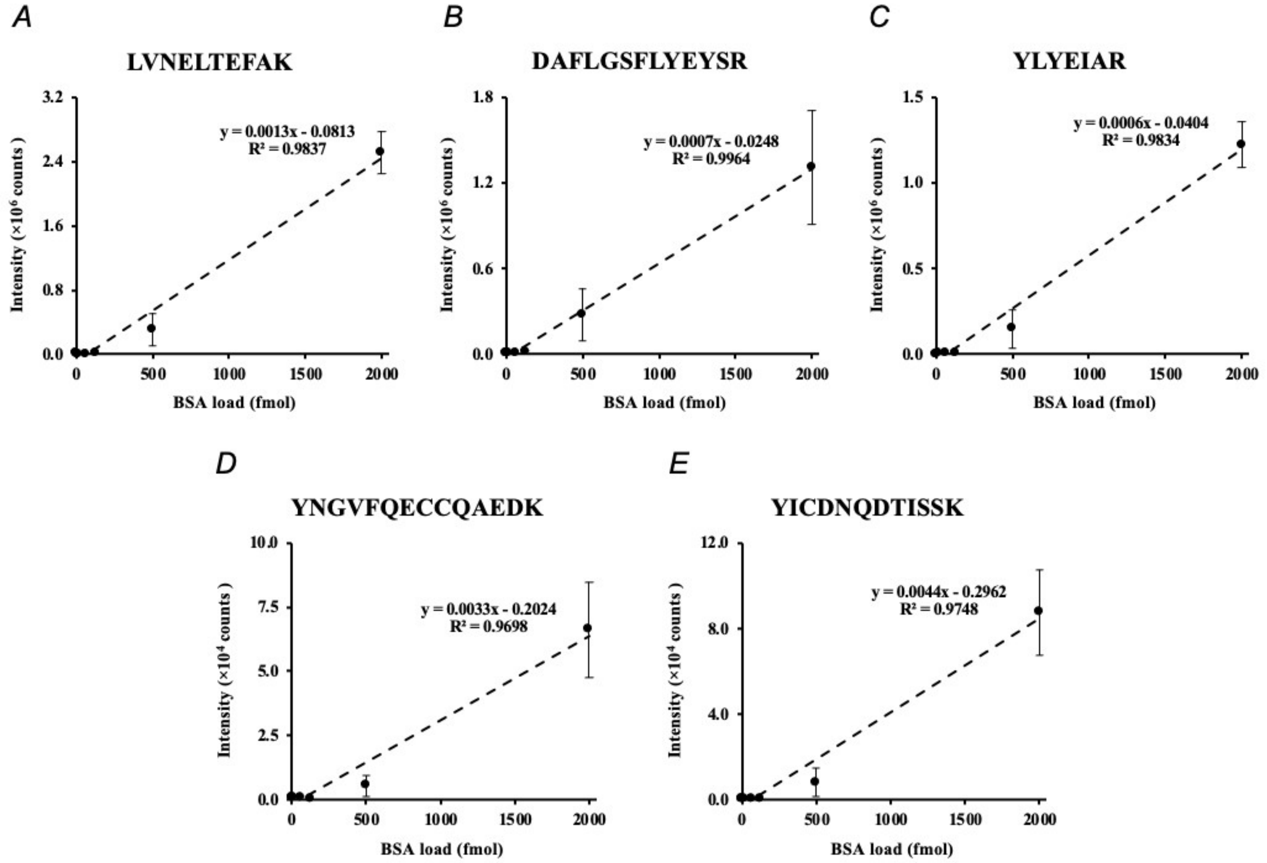

Figure S1-2 Linear regression curves obtained for individual BSA tryptic peptides selected for targeted quantification of BSA in the tryptic hydrolysates of the pea seed protein extracts spiked with different amounts of BSA by selected reaction monitoring (SRM): LVNELTEFAK (A), DAFLGSFLYEYSR (B), YLYEIAR (C), YNGVFQECCQAEDK (D), YICDNQDTISSKGWGPDYPR (E).

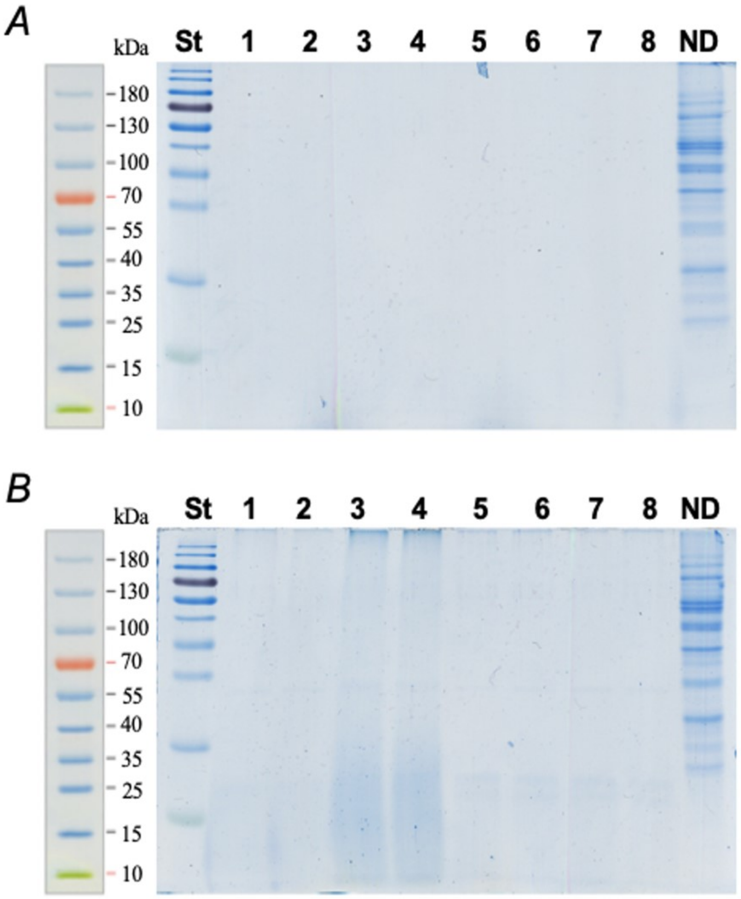

Figure S1-3 SDS-PAGE electrophoreograms acquired for the co-extracted Arabidopsis leaf and pea seed proteins digested with trypsin using the FASP approach (in total 50 µg of protein were applied to each filter unit). The analyses were performed with filtrate (A) and the fraction retained on the filter (B) after 3 x washing with 40 µL of 50 mmol/L aq. NH_4HCO_3 and centrifugal filtration (14 000 g, 10 min). To assess the completeness of hydrolysis, 5µg of each digest (filtrate) were applied on the gel. The whole retained fraction (corresponding to 50 µg of digested protein) was completely transferred to a polypropylene tube, lyophilized, reconstituted in SDS-PAGE sample buffer and loaded on the gel. The overall lane densities were compared to those of non-digested (ND) protein (5 µg) applied to a separate lane. 1-4 – protein isolated with treatment with SDS-containing extraction solution, 5-8 – protein isolated with phenol extraction procedure, St – Page Ruler Prestained Protein Ladder.

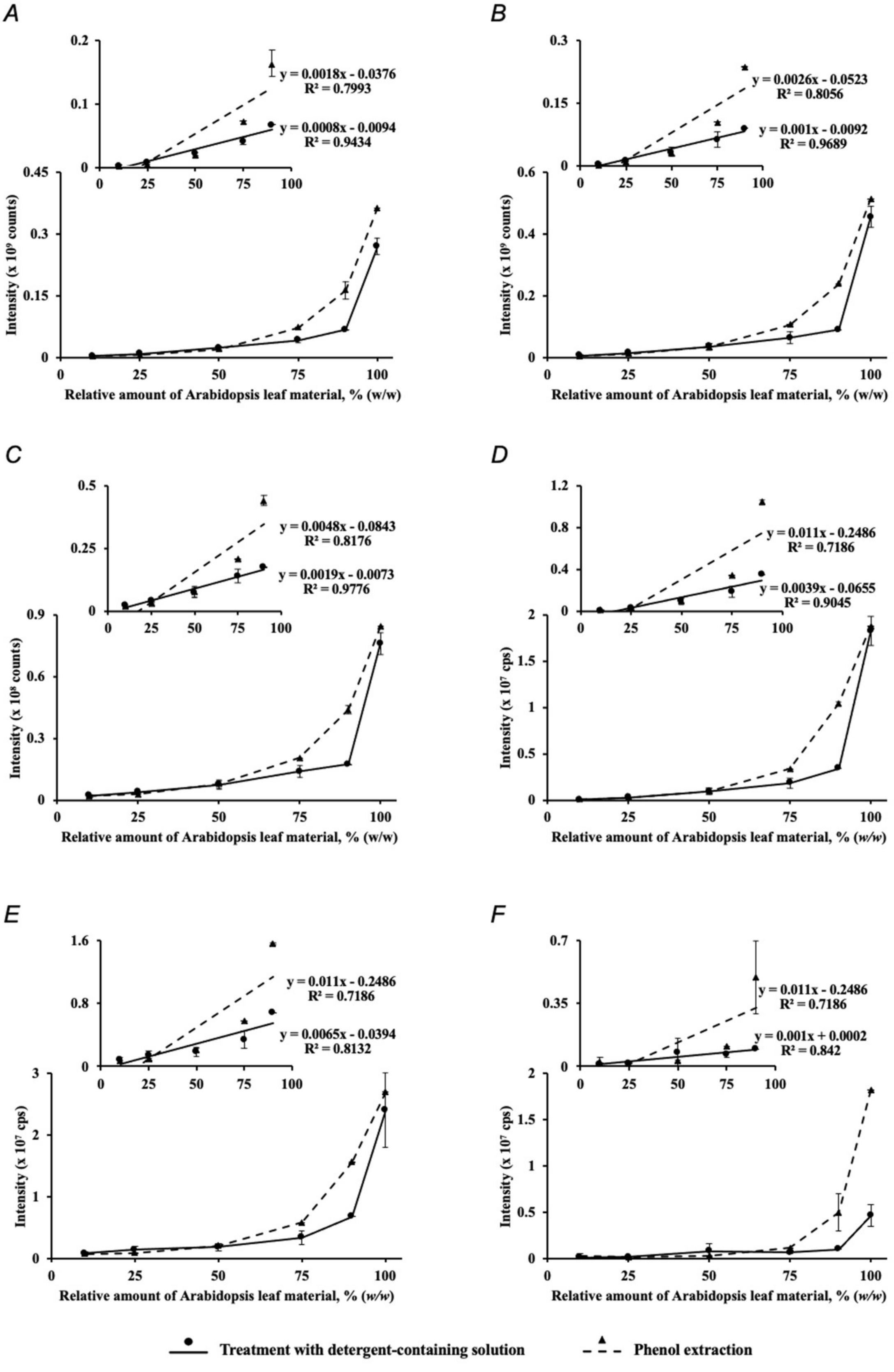
A
y = 0.0018x - 0.0376
R² = 0.7993
y = 0.0008x - 0.0094
R² = 0.9434
Intensity (x 10⁹ counts)
Relative amount of Arabidopsis leaf material, % (w/w)
B
y = 0.0026x - 0.0523
R² = 0.8056
y = 0.001x - 0.0092
R² = 0.9689
Intensity (x 10⁹ counts)
Relative amount of Arabidopsis leaf material, % (w/w)
C
y = 0.0048x - 0.0843
R² = 0.8176
y = 0.0019x - 0.0073
R² = 0.9776
Intensity (x 10⁸ counts)
Relative amount of Arabidopsis leaf material, % (w/w)
D
y = 0.011x - 0.2486
R² = 0.7186
y = 0.0039x - 0.0655
R² = 0.9045
Intensity (x 10⁷ cps)
Relative amount of Arabidopsis leaf material, % (w/w)
E
y = 0.011x - 0.2486
R² = 0.7186
y = 0.0065x - 0.0394
R² = 0.8132
Intensity (x 10⁷ cps)
Relative amount of Arabidopsis leaf material, % (w/w)
F
y = 0.011x - 0.2486
R² = 0.7186
y = 0.001x + 0.0002
R² = 0.842
Intensity (x 10⁷ cps)
Relative amount of Arabidopsis leaf material, % (w/w)
Treatment with detergent-containing solution
Phenol extraction

Figure S1-4 Assessment of the method linearity for quantification of Arabidopsis leaf proteins ribulose-1,5-bisphosphate carboxylase-oxygenase (RuBisCO, A-C) and RuBisCO activase (D-F) after co-extraction from Arabidopsis leaf material added to pea seed powder at the different percentage concentration (10, 25, 50, 75, 90, 100% (*w/w*)) using the phenol extraction procedure or treatment with SDS-containing extraction solution. Quantification of RuBisCO relied on the integrated peak areas obtained for *m/z* 511.2693 ± 0.02, 614.8302 ± 0.02 and 704.3376 ± 0.02 at t_R 60.0, 49.4 and 44.6, corresponding to the $[M+2H]^{2+}$ ions of the proteotypic tryptic peptides DTDILAAFR (A), DLAVEGNEIIR (B) and LTYYTPEYETK (C), respectively, whereas RuBisCO activase was quantified with *m/z* 504.2741 ± 0.02, 849.3843 ± 0.02 and 576.8606 ± 0.02 at t_R 42.2, 40.9 and 67.9, corresponding to the $[M+2H]^{2+}$ ions of the proteotypic tryptic peptides FVESLGVEK (D), GLAYDTSDDQQDITR (E) and VPLILGIWGGK (F), respectively. The peak integration was accomplished in the Quan Browser application of the Xcalibur software (Thermo Fisher Scientific) using the settings listed in the material and method part.

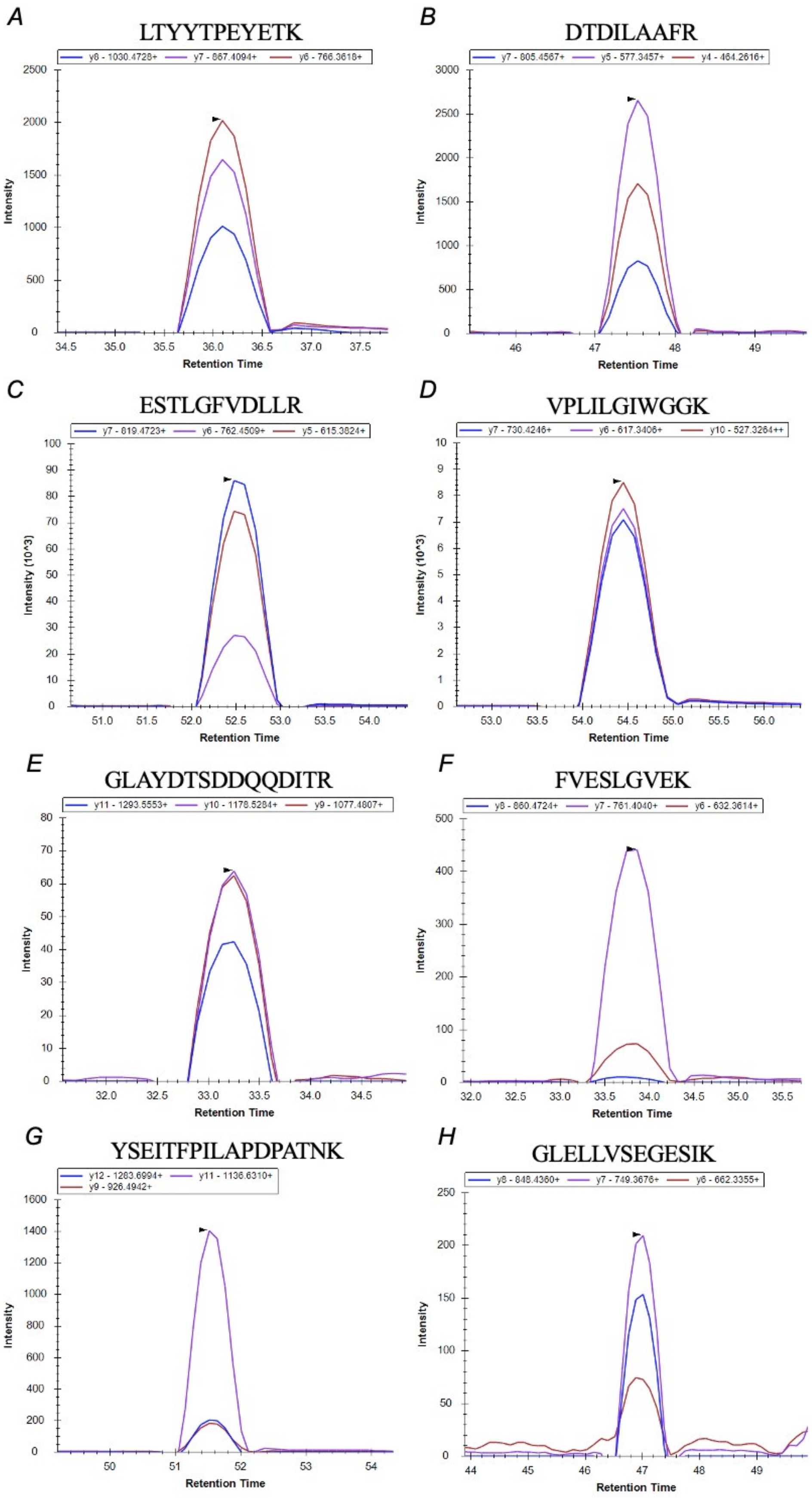
A
LTYYTPEYETK
y8 - 1030.4728+
y7 - 867.4094+
y6 - 766.3618+
Intensity
Retention Time
B
DTDILAAFR
y7 - 805.4567+
y5 - 577.3457+
y4 - 464.2616+
Intensity
Retention Time
C
ESTLGFVDLLR
y7 - 819.4723+
y6 - 762.4509+
y5 - 615.3824+
Intensity (10^3)
Retention Time
D
VPLILGIWGGK
y7 - 730.4246+
y6 - 617.3406+
y10 - 527.3264++
Intensity (10^3)
Retention Time
E
GLAYDTSDDQQDITR
y11 - 1293.5553+
y10 - 1178.5284+
y9 - 1077.4807+
Intensity
Retention Time
F
FVESLGVEK
y8 - 860.4724+
y7 - 761.4040+
y6 - 632.3614+
Intensity
Retention Time
G
YSEITFPILAPDPATNK
y12 - 1283.6994+
y11 - 1136.6310+
y9 - 926.4942+
Intensity
Retention Time
H
GLELLVSEGESIK
y8 - 848.4360+
y7 - 749.3676+
y6 - 662.3355+
Intensity
Retention Time

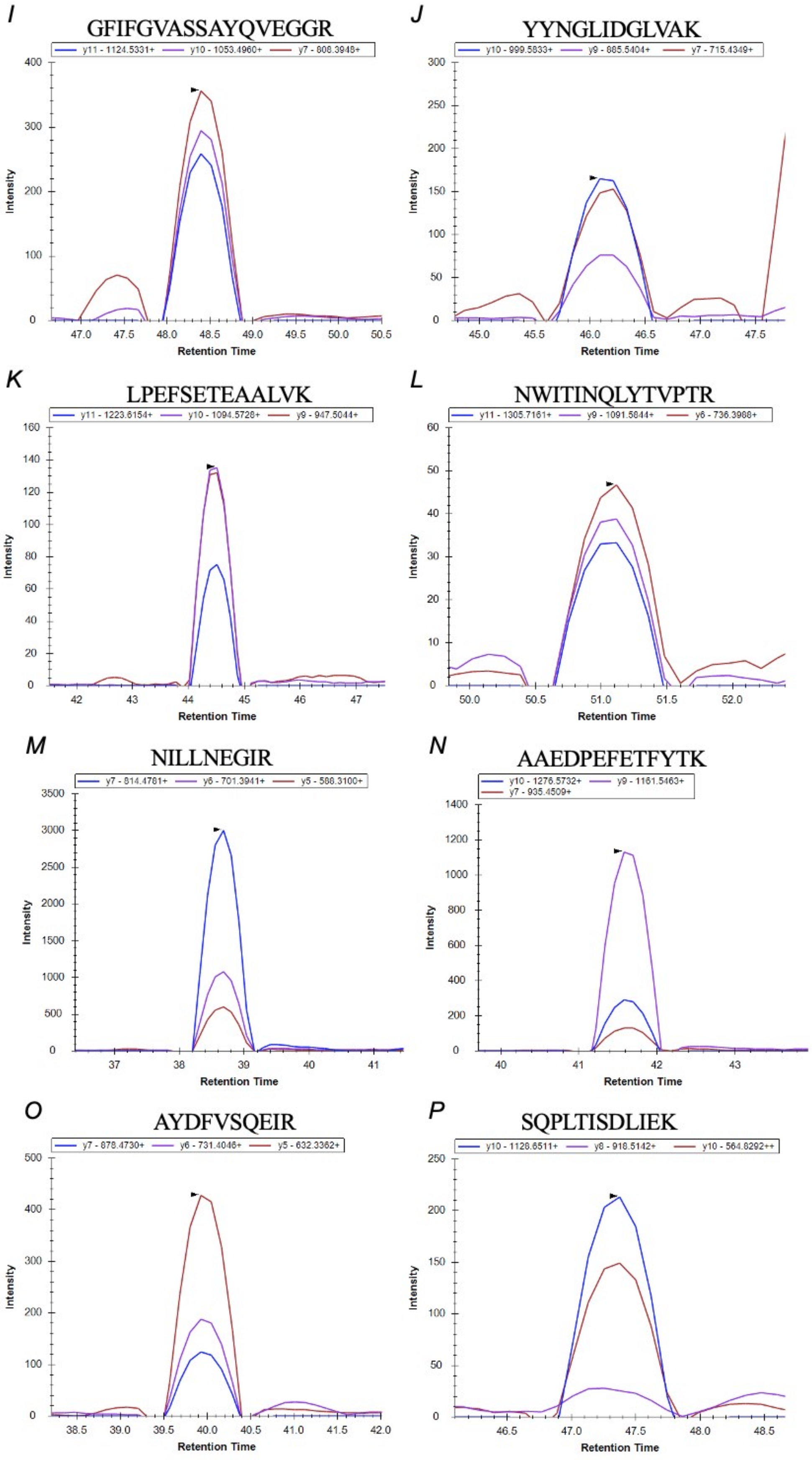
I
GFIFGVASSAYQVEGGR
y11 - 1124.5331+
y10 - 1053.4960+
y7 - 808.3948+
Intensity
Retention Time
J
YYNGLIDGLVAK
y10 - 999.5833+
y9 - 885.5404+
y7 - 715.4349+
Intensity
Retention Time
K
LPEFSETEAALVK
y11 - 1223.6154+
y10 - 1094.5728+
y9 - 947.5044+
Intensity
Retention Time
L
NWITINQLYTVPTR
y11 - 1305.7161+
y9 - 1091.5844+
y6 - 736.3988+
Intensity
Retention Time
M
NILLNEGIR
y7 - 814.4761+
y6 - 701.3941+
y5 - 588.3100+
Intensity
Retention Time
N
AAEDPEFETFYTK
y10 - 1276.5732+
y9 - 1161.5463+
y7 - 935.4509+
Intensity
Retention Time
O
AYDFVSQEIR
y7 - 878.4730+
y6 - 731.4046+
y5 - 632.3362+
Intensity
Retention Time
P
SQPLTISDLIEK
y10 - 1128.6511+
y8 - 918.5142+
y10 - 564.8292++
Intensity
Retention Time

Figure S1-5 Overlays of the extracted ion chromatograms (XICs) obtained for individual fragment ions (specified in the top of each panel) of proteotypic peptides selected for targeted SRM quantification of RuBisCO large chain (LTYYTPEYETK, **A**, DTDILAAFR, **B**, ESTLGFVDLLR, **C**), chloroplastic RuBisCO activase (VPLILGIWGGK, **D**, GLAYDTSDDQQDITR, **E**, FVESLGVEK, **F**), cytochrome f (YSEITFPILAPDPATNK, **G**, GLELLVSEGESIK, **H**), myrosinase 1 (GFIFGVASSAYQVEGGR, **I**, YYNGLIDGLVAK, **J**, LPEFSETEAALVK, **K**, NWITINQLYTVPTR, **L**), photosystem II D2 protein (NILLNEGIR, **M**, AAEDPEFETFYTK, **N**, AYDFVSQEIR, **O**), chloroplastic protein PLASTID TRANSCRIPTIONALLY ACTIVE 16 (SQPLTISDLIEK, **P**). Chromatograms were visualized with Skyline 22.2.0.225 software.

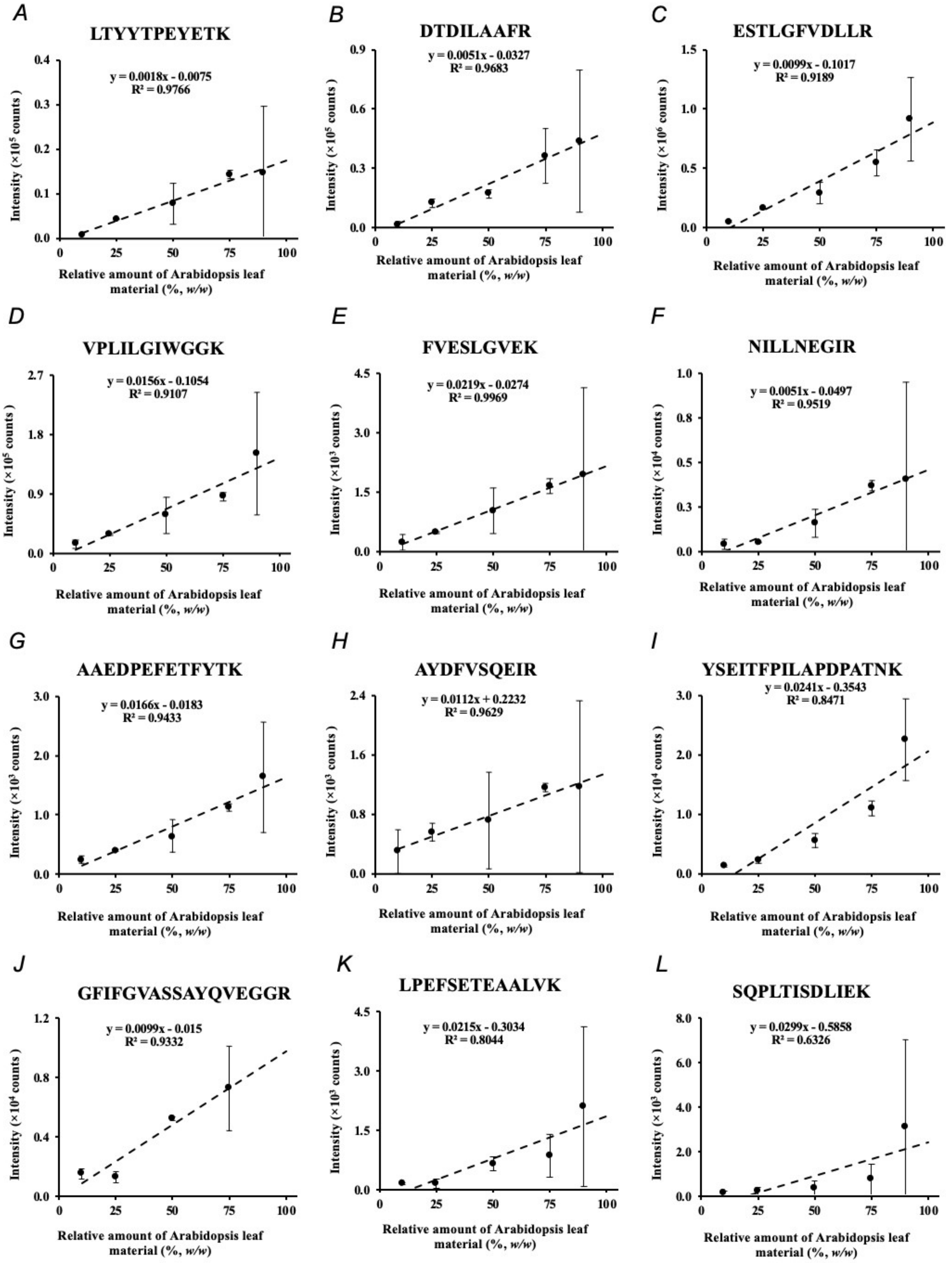
A
LTYYTPEYETK
y = 0.0018x - 0.0075
R^2 = 0.9766
Intensity ($\times 10^5$ counts)
B
DTDILAAFR
y = 0.0051x - 0.0327
R^2 = 0.9683
Intensity ($\times 10^5$ counts)
C
ESTLGFVDLLR
y = 0.0099x - 0.1017
R^2 = 0.9189
Intensity ($\times 10^6$ counts)
D
VPLILGIWGGK
y = 0.0156x - 0.1054
R^2 = 0.9107
Intensity ($\times 10^5$ counts)
E
FVESLGVEK
y = 0.0219x - 0.0274
R^2 = 0.9969
Intensity ($\times 10^3$ counts)
F
NILLNEGIR
y = 0.0051x - 0.0497
R^2 = 0.9519
Intensity ($\times 10^4$ counts)
G
AAEDPEFETFYTK
y = 0.0166x - 0.0183
R^2 = 0.9433
Intensity ($\times 10^3$ counts)
H
AYDFVSQEIR
y = 0.0112x + 0.2232
R^2 = 0.9629
Intensity ($\times 10^3$ counts)
I
YSEITFPILAPDPATNK
y = 0.0241x - 0.3543
R^2 = 0.8471
Intensity ($\times 10^4$ counts)
J
GFIFGVASSAYQVEGGR
y = 0.0099x - 0.015
R^2 = 0.9332
Intensity ($\times 10^4$ counts)
K
LPEFSETEAALVK
y = 0.0215x - 0.3034
R^2 = 0.8044
Intensity ($\times 10^3$ counts)
L
SQPLTISDLIEK
y = 0.0299x - 0.5858
R^2 = 0.6326
Intensity ($\times 10^3$ counts)
Relative amount of Arabidopsis leaf material (%, w/w)

Figure S1-6 Linear regression curves obtained for individual RuBisCO large chain (DTDILAAFR, **A**, LTYYTPEYETK, **B**, ESTLGFVDLLR, **C**), chloroplastic RuBisCO activase (VPLILGIWGGK, **D**, FVESLGVEK, **E**), photosystem II D2 protein (NILLNEGIR, **F**, AAEDPEFETFYTK, **G**, AYDFVSQEIR, **H**), cytochrome f (YSEITFPILAPDPATNK, **I**), myrosinase 1 (GFIFGVASSAYQVEGGR, **J**, LPEFSETEAALVK, **K**), chloroplastic protein PLASTID TRANSCRIPTIONALLY ACTIVE 16 (SQPLTISDLIEK, **L**) tryptic peptides selected for targeted SRM-based quantification of these proteins in the tryptic hydrolysates obtained after the co-extraction of the mixture of Arabidopsis leaf and pea (*P. sativum*) seed material and co-digestion of the resulted protein isolate by the detergent-based.

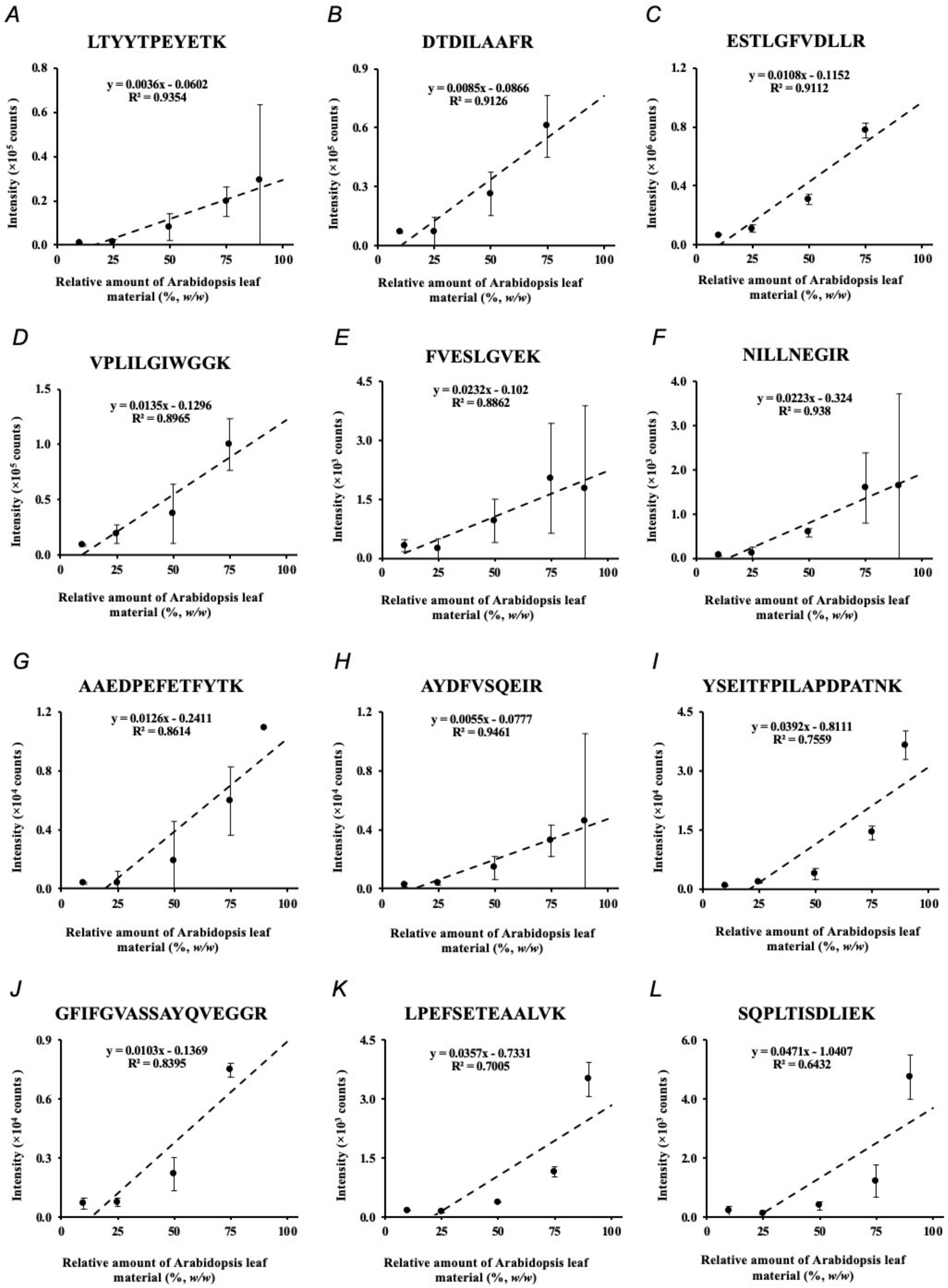
A
LTYYTPEYETK
y = 0.0036x - 0.0602
R² = 0.9354
Intensity (×10⁵ counts)
B
DTDILAAFR
y = 0.0085x - 0.0866
R² = 0.9126
Intensity (×10⁵ counts)
C
ESTLGFVDLLR
y = 0.0108x - 0.1152
R² = 0.9112
Intensity (×10⁶ counts)
D
VPLILGIWGGK
y = 0.0135x - 0.1296
R² = 0.8965
Intensity (×10⁵ counts)
E
FVESLGVEK
y = 0.0232x - 0.102
R² = 0.8862
Intensity (×10³ counts)
F
NILLNEGIR
y = 0.0223x - 0.324
R² = 0.938
Intensity (×10³ counts)
G
AAEDPEFETFYTK
y = 0.0126x - 0.2411
R² = 0.8614
Intensity (×10⁴ counts)
H
AYDFVSQEIR
y = 0.0055x - 0.0777
R² = 0.9461
Intensity (×10⁴ counts)
I
YSEITFPILAPDPATNK
y = 0.0392x - 0.8111
R² = 0.7559
Intensity (×10⁴ counts)
J
GFIFGVASSAYQVEGGR
y = 0.0103x - 0.1369
R² = 0.8395
Intensity (×10⁴ counts)
K
LPEFSETEAALVK
y = 0.0357x - 0.7331
R² = 0.7005
Intensity (×10³ counts)
L
SQPLTISDLIEK
y = 0.0471x - 1.0407
R² = 0.6432
Intensity (×10³ counts)
Relative amount of Arabidopsis leaf material (%, w/w)

Figure S1-7 Linear regression curves obtained for individual RuBisCO large chain (DTDILAAFR, **A**, LTYYTPEYETK, **B**, ESTLGFVDLLR, **C**), chloroplastic RuBisCO activase (VPLILGIWGGK, **D**, FVESLGVEK, **E**), photosystem II D2 protein (NILLNEGIR, **F**, AAEDPEFETFYTK, **G**, AYDFVSQEIR, **H**), cytochrome f (YSEITFPILAPDPATNK, **I**), myrosinase 1 (GFIFGVASSAYQVEGGR, **J**, LPEFSETEAALVK, **K**), chloroplastic protein PLASTID TRANSCRIPTIONALLY ACTIVE 16 (SQPLTISDLIEK, **L**) tryptic peptides selected for targeted SRM-based quantification of these proteins in the tryptic hydrolysates obtained after the co-extraction of the mixture of Arabidopsis leaf and pea (*P. sativum*) seed material and co-digestion of the resulted protein isolate by the phenol-based.

Tables

Table S1-1 Proteotypic BSA tryptic peptides selected for targeted quantification of BSA by selected reaction monitoring in the tryptic hydrolyzates of the pea seed protein extracts spiked with different amounts of BSA

Target peptide sequence	$[M+H]^{2+}$ (*m/z*)	Fragment 1 (*m/z*)	Fragment 2 (*m/z*)	Fragment 3 (*m/z*)
YNGVFQECCQAEDK	874	1315	1039	910
DAFLGSFLYEYSR	784	1122	1235	717
YICDNQDTISSK	722	1167	1007	650
LVNELTEFAK	582	951	708	595
YLYEIAR	464	651	488	359

The analysis relied on nonscheduled SRM acquisition method for quantitation of up to 10 peptides in one run (three transitions per peptide) using an Orbitrap XL hybrid mass spectrometer equipped with a NanoFlex source, coupled online to an Ultimate 3000 nano-HPLC system (Thermo Fisher Scientific, Bremen, Germany). Mass tolerances for searching precursor ion and fragment ions were defined as trap isolation windows and accounted ± 0.5 and 1.5 Da, respectively.

Table S1-2 Sensitivity and linearity data acquired for individual BSA tryptic peptides in the spike with pea seed protein hydrolyzate employed in the targeted quantification experiments with spiked pea seed protein

**Target peptide sequence**	**t_R, min**	**$[M+H]^{2+}$ (*m/z*)**	**LOD (fmol)**	**LOQ (fmol)**	**LDR**
YNGVFQECCQAEDK	34.5	874	125	500	0.15×10^2
DAFLGSFLYEYSR	57.5	784	4	125	0.6×10^2
YICDNQDTISSK	29.1	722	125	500	0.15×10^2
LVNELTEFAK	43.1	582	16	125	0.6×10^2
YLYEIAR	37.6	464	62.5	125	0.6×10^2

BSA (50 µL of 6.6×10^{-5} - 1.32×10^{-1} g/L solution) was spiked to 8 – 9.2 µL of the pea seed protein with the total protein concentration of 5.45 g/L, adjusted up to 200 µL with urea solution. BDL, below detection limit.

Table S1-3 Number of protein groups, proteins, and individual peptides of *Arabidopsis thaliana* identified after two protein isolation protocols

**Protein extraction protocol**	**# Protein groups**	**# Proteins**	**# Peptides**
Phenol extraction	538	1161	1613
Detergent treatment	711	1397	1843

Table S1-4 Number of membrane proteins of *Arabidopsis thaliana* analyzed after two protein isolation protocols

Protein extraction protocol	TM	SP + TM	SP	GLOB	BETA
Phenol extraction	130	18	183	1078	2
Detergent treatment	120	16	92	945	5

TM – alpha helical transmembrane proteins without a signal peptide, SP + TM – alpha helical transmembrane proteins with signal peptide, SP – proteins with signal peptides, BETA – beta-barrel transmembrane proteins, GLOB – globular proteins without signal peptide

Table S1-5 Number of protein regions of Arabidopsis thaliana analyzed after two protein isolation protocols

**Protein extraction protocol**	**TMhelix**	**signal**	**inside**	**outside**	**TMbeta**
Phenol extraction	449	203	1375	501	36
Detergent treatment	536	113	1302	453	92

Table S1-6 Proteotypic tryptic peptides of Arabidopsis proteins selected for targeted quantification by selected reaction monitoring in the samples co-extracted and co-digested with pea seed proteins

Protein description	Peptide sequence	[M+H]$^{2+}$ (*m/z*)	Fragment 1 (*m/z*)	Fragment 2 (*m/z*)	Fragment 3 (*m/z*)
Ribulose bisphosphate carboxylase large chain	DTDILAAFR	511	577	805	464
	LTYYTPEYETK	704	766	867	1030
	ESTLGFVDLLR	625	819	762	615
RuBisCO activase	VPLILGIWGGK	577	528	617	730
	GLAYDTSDDQQDITR	849	1077	1179	1293
	FVESLGVEK	504	761	632	860
Photosystem II D2 protein	NILLNEGIR	521	814	701	588
	AAEDPEFETFYTK	774	1161	1276	935
	AYDFVSQEIR	614	632	731	878
Cytochrome f	SNNTVYNATAGGIISK	805	1095	931	1194
	YSEITFPILAPDPATNK	939	1137	1284	927
	GLELLVSEGESIK	687	749	848	662

Protein PLASTID TRANSCRIPTIONALLY ACTIVE 16, chloroplastic	LGSQFATAIQNASETPK	882	987	1160	1059
	SQPLTISDLIEK	672	1129	565	918
	LNAVQSPFQDAESIAK	859	1193	1106	1321
Myrosinase 1	GFIFGVASSAYQVEGGR	873	808	1053	1124
	YYNGLIDGLVAK	663	1000	715	886
	LPEFSETEAALVK	717	947	1095	1224
	NWITINQLYTVPTR	860	1092	736	1306

The analysis relied on nonscheduled SRM acquisition method quantifying up to 10 peptides in one run, three transitions per peptide, using an Orbitrap XL hybrid mass spectrometer equipped with a NanoFlex source, coupled online to an Ultimate 3000 nano-HPLC system (Thermo Fisher Scientific, Bremen, Germany). Mass tolerances for searching precursor ion and fragment ions were ± 0.5 and 1.5 Da, respectively.

Table S1-7 Sensitivity and linearity data acquired for individual tryptic peptides of Arabidopsis proteins employed in the targeted quantification experiments in the samples co-extracted and co-digested (by the detergent-based protocol) with pea seed proteins

Protein description	Peptide sequence	t_R, min	$[M+H]^{2+}$ (m/z)	LOD (% of Arabidopsis material)	LOQ (% of Arabidopsis material)	LDR
Ribulose bisphosphate carboxylase large chain	DTDILAAFR	47.5	511	10	10	0.09×10^2
	LTYYTPEYETK	36	704	10	25	0.04×10^2
	ESTLGFVDLLR	52.4	625	10	10	0.09×10^2
Ribulose bisphosphate carboxylase/oxygenase activase, chloroplastic	VPLILGIWGGK	54.3	577	10	10	0.09×10^2
	GLAYDTSDDQQDITR	33.1	849	100	-	-
	FVESLGVEK	33.2	504	10	25	0.04×10^2
Photosystem II D2 protein	NILLNEGIR	38.2	521	10	10	0.09×10^2
	AAEDPEFETFYTK	41.2	774	10	10	0.09×10^2
	AYDFVSQEIR	39.4	614	10	25	0.04×10^2
Cytochrome f	SNNTVYNATAGGIISK	-	805	-	-	-
	YSEITFPILAPDPATNK	51.5	939	10	25	0.04×10^2

	GLELLVSE GESIK	46.9	687	100	-	-
Protein PLASTID TRANSCRIPTIONALLY ACTIVE 16, chloroplastic	LGSQFAT AIQNASET PK	-	882	-	-	-
	SQPLTISD LIEK	47.3	672	50	75	0.01×10^2
	LNAVQSP FQDAESIA K	-	859	-	-	-
Myrosinase 1	GFIFGVAS SAYQVEG GR	48.3	873	10	25	0.04×10^2
	YYNGLID GLVAK	46.1	663	90	-	-
	LPEFSETE AALVK	44.4	717	25	75	0.01×10^2
	NWITINQL YTVPTR	50.9	860	90	-	-

Table S1-8 Sensitivity and linearity data acquired for individual tryptic peptides of Arabidopsis proteins employed in the targeted quantification experiments in the samples co-extracted and co-digested (by the phenol extraction protocol) with pea seed proteins

Protein description	Peptide sequence	t_R, min	$[M+H]^{2+}$ (*m/z*)	LOD (% of Arabidopsis material)	LOQ (% of Arabidopsis material)	LDR
Ribulose bisphosphate carboxylase large chain	DTDILAAF R	47.5	511	10	10	0.09 x 10^2
	LTYYTPE YETK	36	704	10	10	0.09 x 10^2
	ESTLGFV DLLR	52.4	625	10	10	0.09 x 10^2
Ribulose bisphosphate carboxylase/oxygenase activase, chloroplastic	VPLILGIW GGK	54.3	577	10	10	0.09 x 10^2
	GLAYDTS DDQQDIT R	33.1	849	100	-	-
	FVESLGV EK	33.2	504	10	25	0.04 x 10^2
Photosystem II D2 protein	NILLNEGI R	38.2	521	10	10	0.09 x 10^2
	AAEDPEF ETFYTK	41.2	774	10	25	0.04 x 10^2
	AYDFVSQ EIR	39.4	614	10	25	0.04 x 10^2
Cytochrome f	SNNTVYN ATAGGIIS K	-	805	-	-	-
	YSEITFPIL APDPATN K	51.5	939	10	25	0.04 x 10^2
	GLELLVSE	46.9	687	90	-	-

	GESIK					
Protein PLASTID TRANSCRIPTIONALLY ACTIVE 16, chloroplastic	LGSQFATAIQNASETPK	-	882	-	-	-
	SQPLTISDLIEK	47.3	672	75	75	0.01×10^2
	LNAVQSPFQDAESIAK	-	859	-	-	-
Myrosinase 1	GFIFGVASSAYQVEGGR	48.3	873	25	25	0.04×10^2
	YYNGLIDGLVAK	46.1	663	90	-	-
	LPEFSETEAALVK	44.4	717	50	75	0.01×10^2
	NWITINQLYTVPTR	50.9	860	90	-	-
